# Interindividual Variability of Lower-Limb Motor Cortical Plasticity Induced by Theta Burst Stimulation

**DOI:** 10.3389/fnins.2020.563293

**Published:** 2020-11-13

**Authors:** Natsuki Katagiri, Shinya Yoshida, Tadaki Koseki, Daisuke Kudo, Shigehiro Namba, Shigeo Tanabe, Ying-Zu Huang, Tomofumi Yamaguchi

**Affiliations:** ^1^Department of Physical Therapy, Yamagata Prefectural University of Health Sciences, Yamagata, Japan; ^2^Faculty of Rehabilitation, School of Health Sciences, Fujita Health University, Aichi, Japan; ^3^Neuroscience Research Center and Department of Neurology, Chang Gung Memorial Hospital and Chang Gung University College of Medicine, Taoyuan, Taiwan; ^4^Department of Physical Therapy, Faculty of Health Science, Juntendo University, Tokyo, Japan

**Keywords:** non-invasive brain stimulation, cortical plasticity, interindividual variability, primary motor cortex, lower-limb, transcranial magnetic stimulation

## Abstract

Theta burst stimulation (TBS) has been used as a tool to induce synaptic plasticity and improve neurological disorders. However, there is high interindividual variability in the magnitude of the plastic changes observed after TBS, which hinders its clinical applications. The electric field induced by transcranial magnetic stimulation (TMS) is strongly affected by the depth of the stimulated brain region. Therefore, it is possible that the variability in the response to TBS over the lower-limb motor cortex is different for the hand area. This study investigated the variability of TBS-induced synaptic plasticity in the lower-limb motor cortex, for intermittent TBS (iTBS), continuous TBS (cTBS), and sham iTBS, in 48 healthy young participants. The motor cortical and intracortical excitability of the tibialis anterior was tested before and after TBS using TMS. The results showed that iTBS had facilitatory effects on motor cortex excitability and intracortical inhibition, whereas cTBS exerted opposite effects. Twenty-seven percent of individuals exhibited enhanced motor cortical plasticity after iTBS, whereas 63% of participants showed enhanced plasticity after cTBS. In addition, the amount of TBS-induced plasticity was correlated with the intracortical excitability and the variability of the motor evoked potential prior to TBS. Our study demonstrated the high variability of the iTBS-induced lower-limb motor cortical plasticity, which was affected by the sensitivity of intracortical interneuronal circuits. These findings provide further insights into the variation of the response to TBS according to the anatomy of the stimulated brain region and the excitability of the intracortical circuit.

## Introduction

Theta burst stimulation (TBS) is a non-invasive brain stimulation (NIBS) that can modulate the cortical excitability of the human brain depending on the pulse frequency (Huang et al., 2005). Intermittent TBS (iTBS) has a facilitatory effect on motor cortex excitability up to 30 min, whereas continuous TBS (cTBS) has the opposite effect up to 60 min (Chung et al., 2016). Previous reviews showed that these TBS-induced effects of motor cortex excitability are known as long-term potentiation (LTP)-like and long-term depression (LTD)-like synaptic plasticity, respectively (Suppa et al., 2016; Huang et al., 2017).

The inter- and intra-individual variability of cortical plasticity after TBS is a key issue of this tool (Hamada et al., 2013; Hinder et al., 2014; López-Alonso et al., 2014; Hordacre et al., 2017; Sasaki et al., 2018). The variability of the TBS-induced plasticity hinders its clinical applications as a potential therapy for neurological disorders (Terranova et al., 2019). Several factors contribute to the variation that occurs in response to TBS, such as biological factors including age, genetics, sex, and anatomy of the neural circuits (Suppa et al., 2016; Huang et al., 2017).

iTBS selectivity enhances the late indirection wave (I-wave) originating from layer 2 and 3 neurons, while cTBS suppresses the I1-wave originating from monosynaptic connections to layer 5 pyramidal neurons (Di Lazzaro and Rothwell, 2014; Suppa et al., 2016). These reports highlight the fact that each TBS protocol can modulate different cortical circuits. Moreover, it has been proposed that the recruitment of late I-wave-generating circuits predicts the individual induction of synaptic plasticity that occurs after TBS (Hamada et al., 2013; Volz et al., 2019). In addition, the transcranial magnetic stimulation (TMS)-induced electric field strongly depends on the depth of the stimulated brain region and the direction of the sulcus (Laakso et al., 2014). Thus, if the strength of the TBS-induced electric field changes according to the depth of the representation of the motor cortex and/or the direction of the sulcus, the aftereffects of TBS might vary.

Lower-limb representation has a deeper position in the motor cortex compared with the hand area. Moreover, the layer of the motor cortex is located parallel to a sagittal plane, and the bilateral areas are located very close to each other (Huang et al., 2018; Lin et al., 2019). Therefore, it is possible that the induction of an electric field by TBS is different between the lower-limb and hand areas. However, to our knowledge, no studies have addressed the interindividual variability of the lower-limb motor cortex plasticity induced by TBS.

To investigate the variability of TBS-induced synaptic plasticity on lower-limb motor cortex, we used three protocols of TBS (iTBS, cTBS, and sham iTBS) to modulate corticospinal and intracortical excitability. Furthermore, inhibitory or facilitatory interneurons play important roles in the TBS-induced plasticity of the cortical excitability of stimulated regions (Suppa et al., 2016; Li et al., 2019). Thus, we hypothesized that the excitability of intracortical inhibition or facilitation before TBS may be associated with the plastic changes in corticospinal excitability induced by TBS.

## Materials and Methods

### Participants

Forty-eight healthy participants [24 women; 19–27 years of age (mean ± standard deviation, 21.3 ± 2.1 years)] participated in this study who neither had a history of neurological and/or orthopedic diseases nor were being treated with a medication that affected the central nervous system. The sample size was calculated at *n* = 45 or more using a power analysis based on the effect size (g = 0.43) reported by a previous study that investigated the effects of TBS on corticospinal excitability (Chung et al., 2016). According to the foot-preference test (Chapman and Chapman, 1987), 47 participants showed right leg dominance and one showed left leg dominance. The 48 participants were naïve regarding TBS. The participants gave their written informed consent before participation in the study. The study was approved by the Ethics Review Board of the Yamagata Prefectural University of Health Sciences (approval number: 1806-06) and was performed in accordance with the Declaration of Helsinki. This study was registered at the University Hospital Medical Information Network (registration number: 000036852).

### Experimental Procedure

**Figure 1:**
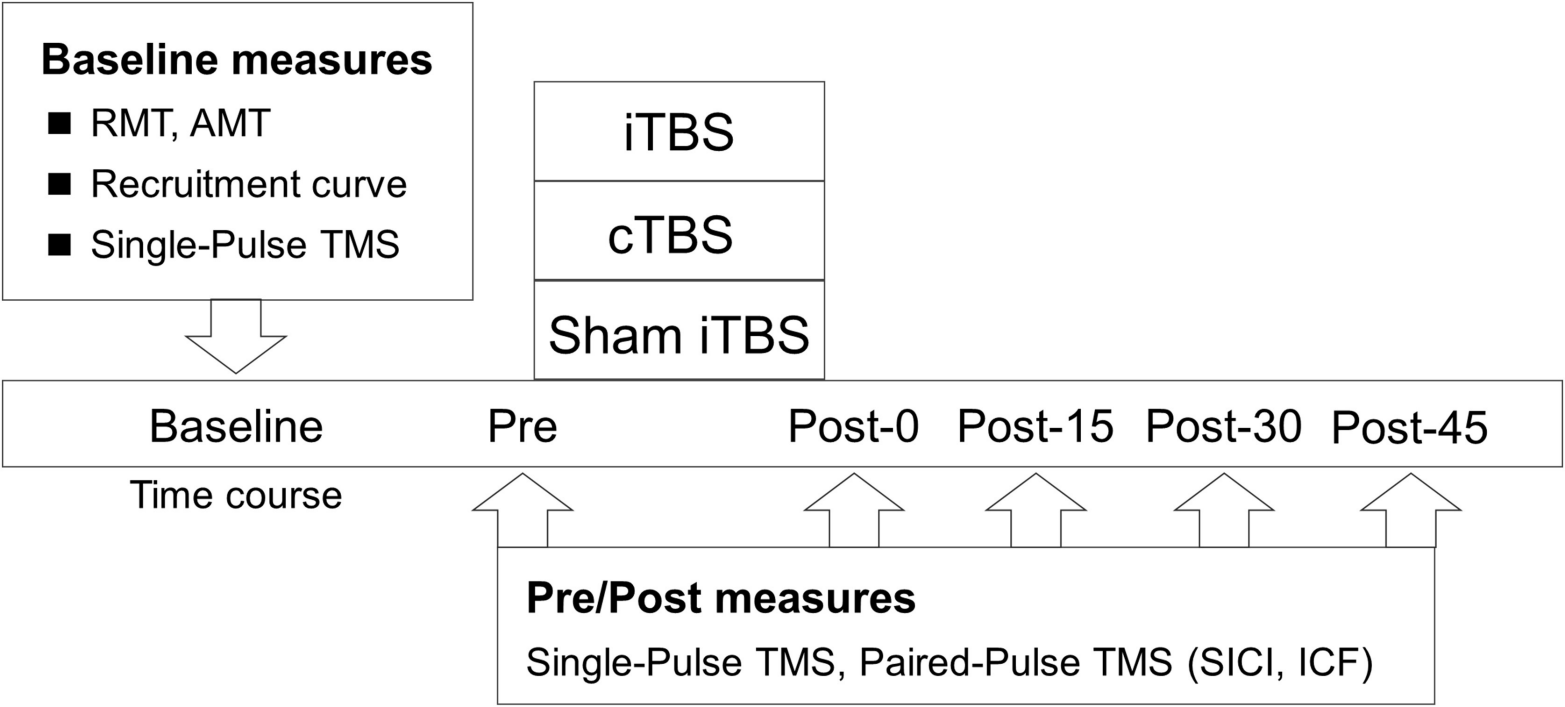
Experimental procedure. The participants received iTBS, cTBS, and sham iTBS randomly at intervals of over 3 days. The resting motor threshold (RMT), active motor threshold (AMT), recruitment curve, and single-pulse TMS were assessed at the baseline. Single- and paired-pulse TMS were assessed before TBS (Pre) and at 0 (Post-0), 15 (Post-15), 30 (Post-30), and 45 min (Post-45) after TBS.

This study had a single-blinded (participants), sham-controlled, crossover experimental design (Figure 1). Forty-eight subjects participated in this study, which consisted of 3 blocks of counterbalanced ordered sessions (iTBS, cTBS, and sham iTBS) across participants, separated by at least 3 days. The following factors, which reportedly affect the effectiveness of NIBS, were controlled in each subject: experimental time, vigorous physical activity, alcohol and caffeine intake, and sleep the day before the experiment (Guerra et al., 2020). Before the assessment of the main outcome, a recruitment curve (RC) was generated to explore the predictors of the variability in the response to TBS. Next, the baseline corticospinal excitability was measured to normalize the motor evoked potential (MEP) data. After the measurements, main neurophysiological assessments [corticospinal excitability, short-interval intracortical inhibition (SICI), and intracortical facilitation (ICF)] were carried out before TBS (Pre) and after TBS, every 15 min for 45 min (Post-0, Post-15, Post-30, and Post-45).

### Theta Burst Stimulation

Each TBS was composed of theta frequency bursts consisting of three stimuli at 50 Hz each. The cTBS protocol was applied as 200 consecutive bursts at 5 Hz (40 s, 600 stimuli); the iTBS protocol was delivered as 10 bursts of 5 Hz every 10 s (190 s, 600 stimuli) (Huang et al., 2005). Each TBS was performed using a MagPro R30 instrument (MagVenture A/S, Denmark) connected to a figure-of-eight coil with an outer diameter of 75 mm (Cool-B65). The coil was placed over the hotspot of the tibialis anterior (TA) muscle. The TBS intensity was 80% of the active motor threshold (AMT). The AMT was defined as the minimum stimulation intensity required to evoke a liminal MEP in the TA muscle (>200 μV in 50% of the 10 trials) while inducing isometric contraction with electromyography (EMG) amplitudes of 100 μV in the TA muscle (Yamaguchi et al., 2018). In consideration of the effects of voluntary contraction on the TBS-induced plasticity, an interval of >10 min was set between the measurement of AMT and the adaptation of TBS (Gentner et al., 2008). For the sham iTBS, iTBS was applied on the same area with the stimulation coil turned over (Huang et al., 2009; Yamaguchi et al., 2018).

### Electromyography

The participants were comfortably seated in a chair with their arms resting on a cushion. The EMG was recorded via Ag/AgCl-plated surface electrodes (diameter, 1 cm) that were placed 2 cm apart over the tested muscles in the right TA muscle. Responses were acquired using a Neuropack MEB-2200 system (Nihon Kohden, Tokyo, JPN) with filters set at 10 Hz and 1 kHz. Signals were recorded at a sampling rate of 5 kHz and stored on the computer for later analysis using the LabVIEW software (National Instruments Inc., Austin, Texas, USA). The EMG activity was monitored online. If the amplitude of background TA EMG exceeded 10 μV, the trial was rejected, and repeated.

### Transcranial Magnetic Stimulation

To assess changes in motor cortex excitability, we applied single-pulse TMS to the leg area of the left primary motor cortex using a double-cone coil connected to a Magstim BiStim2 machine (Magstim Company; Whitland, UK). The hotspot of the primary motor cortex was confirmed based on the induction of maximum and sustained MEP in the TA muscle at rest. This position was marked with a pen on the scalp, for repositioning the coil subsequently. The stimulation intensity was adjusted to 120% of the resting motor threshold (RMT), which was defined as the minimum stimulation intensity over the motor hotspot that was required to evoke a MEP of no <50 μV in 5 out of 10 trials (Rossi et al., 2009). The MEP amplitudes were normalized to the baseline MEP amplitudes (%) for statistical analysis.

We applied a subthreshold conditioning paired-pulse paradigm to test SICI and ICF (Kujirai et al., 1993). We used 80% of the AMT for the conditioning stimulus and 120% of the RMT for the test stimulus. AMT for TMS measurements and TBS were separately assessed using each TMS stimulator. The intensity of the test stimulus was adjusted to maintain the average amplitude recorded before TBS throughout the experiment. The interstimulus intervals (ISIs) were 2.5 ms (SICI) and 10 ms (ICF), and 15 trials were recorded for each ISI and test stimulation. We selected an ISI of 2.5 ms to avoid mixing different mechanisms of SICI (Fisher et al., 2002). The ISIs of TMS were controlled by custom-made LabVIEW scripts. Stimuli were applied as blocks in a random fashion. The conditioned MEP amplitudes at ISIs of 2.5 and 10 ms were expressed as percentages of the mean test MEP amplitudes.

An RC was generated to explore the predictive factors of response to TBS. The TMS intensities were increased by 20% per step, from 80 to 200% of the AMT. Each intensity was applied in a pseudorandom order (Kleim et al., 2007). Participants were asked to keep the muscle contractions at amplitudes of 100 μV with visual feedback from the EMG. Based on the data points obtained, regression plots were fit to the approximately linear part of the RC, and the slope of the RC was calculated (Hardwick et al., 2014). In addition, we calculated the coefficient of variation of the MEP (MEP-CV) according to the following equation: MEP-CV = standard deviation/mean peak-to-peak MEP amplitude. A previous report suggested that MEP-CV was related to the response to cTBS on the hand motor cortex area (Hordacre et al., 2017). For all TMS measurements, 15 stimuli were delivered every 5 s at each time point in pseudorandom timing. The raw wave forms in which muscle contractions over 10 μV were mixed were rejected and re-measured. Any raw waveforms in which the amplitude of background TA EMG were rejected and re-measured. In consideration of the amplitude variability, the first waveform was removed from all the TMS testings. Then, the waveforms that exceeded ± 2 SD calculated from the amplitudes of 14 waveforms were removed. The intensity of TBS, raw RC values, and MEP-CV collected from the individuals for each TBS protocol can be found in the Supplementary Material.

### Statistical Analysis

To determine the effects of each TBS protocol (iTBS, cTBS, and sham iTBS) and time (Pre, Post-0, Post-15, Post-30, and Post-45) on normalized MEP amplitudes, SICI, and ICF, we used a linear mixed model based on two-way repeated-measures analysis of variance (ANOVA). Subjects were included as random effects for all mixed models. When ANOVA showed significant main effects and interactions, further investigations were performed using paired *t*-tests with Bonferroni adjustments for multiple comparisons.

To investigate the interindividual variability in response to each TBS, we performed a two-step cluster analysis (López-Alonso et al., 2014; van de Ruit and Grey, 2019). Most previous studies used a classification based on the average % baseline MEPs obtained immediately after TBS to 30 or 60 min after TBS (Hamada et al., 2013; López-Alonso et al., 2014; Hordacre et al., 2017; Sasaki et al., 2018). However, MEPs at a later time than immediately after TBS may include recovery from the TBS-induced changes (Hulme et al., 2013; Müller-Dahlhaus and Ziemann, 2015). Therefore, normalized MEPs at Post-0 were used for the cluster analysis of dependent variables. The distance and cluster criteria required for the analysis were defined as the Euclidian distance and Bayesian information criterion, respectively. The optimal number of clusters was determined automatically based on the Bayesian information criterion with no limit (Schwartz, 1978; van de Ruit and Grey, 2019). Based on a previous report (López-Alonso et al., 2014), each cluster was classified as “responders” or “non-responders.” In addition, we performed Mann–Whitney *U*-test with Bonferroni correction to examine changes in the corticospinal excitability between “responders” and “non-responders” at Post-0.

We used a binary logistic regression analysis to examine the factors that affected each TBS response in baseline measurements. The dependent variable was the cluster of each TBS and the independent variables were SICI, ICF, MEP-CV, the slope of the RC, RMT, and AMT. However, a previous study has reported that the classification of responders by cluster analysis includes a false positive (van de Ruit and Grey, 2019). Thus, we also used Spearman's rank correlation coefficients for normalized MEP at Post-0 and SICI, ICF, MEP-CV, the slope of the RC, RMT, and AMT to examine the relations between individual differences of TBS-induced plasticity and neurophysiological factors at baseline. To avoid type II statistical errors, we did not adjust the *P*-values. Significance was set at *P* < 0.05. All analyses were performed using IBM SPSS 24.0 (IBM Corp., New York, NY, USA) for Windows.

## Results

### MEPs

**Figure 2:**
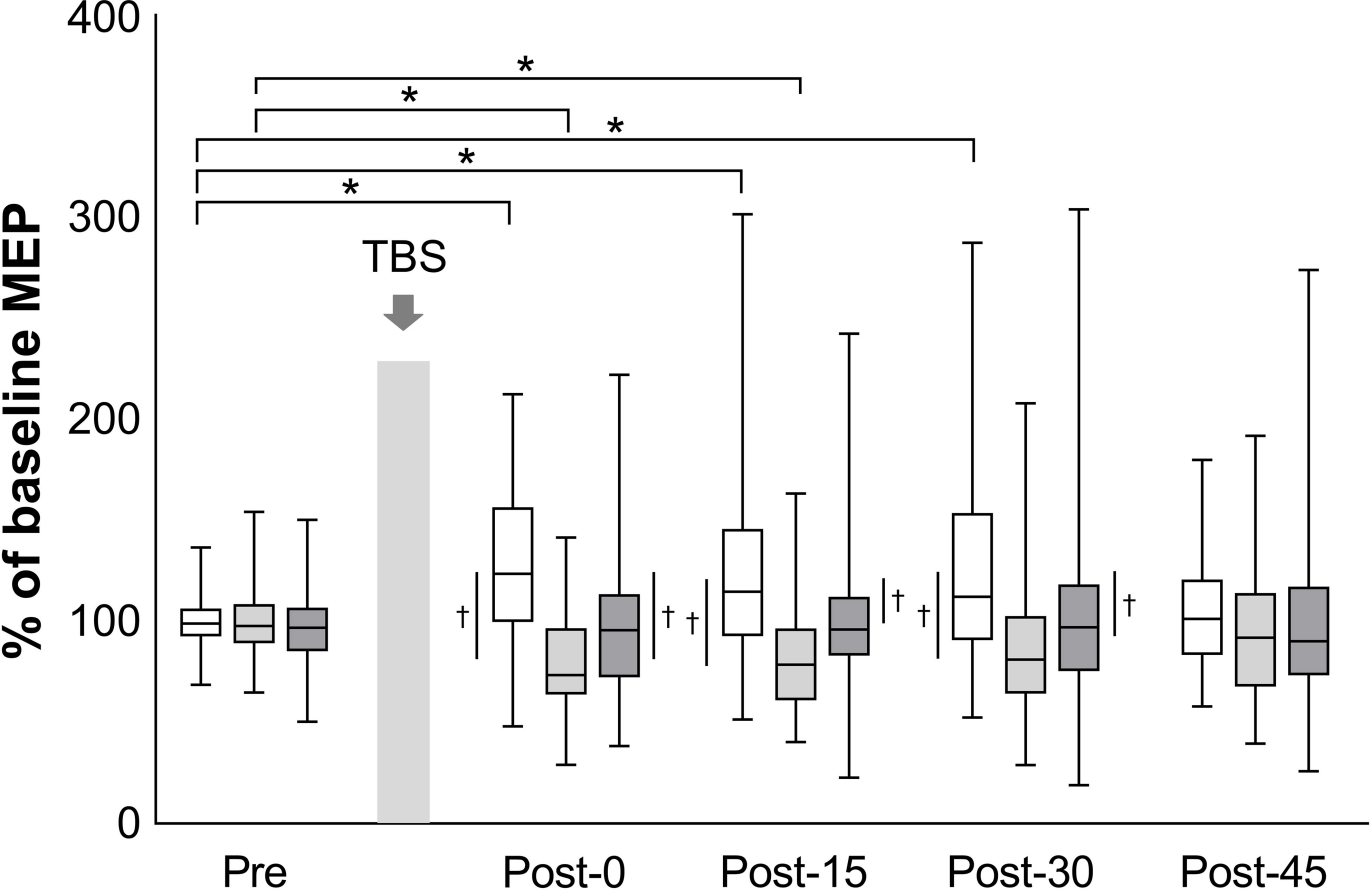
Effects of TBS on corticospinal excitability. The amplitudes of MEP are normalized to the baseline MEP values. White box plots indicate iTBS; light gray, cTBS; dark gray, sham iTBS. Median and interquartile ranges are represented by horizontal lines within boxes and whiskers (representing minimum and maximum values), respectively. The asterisk denotes significant differences between Pre and each time point within the conditions. The dagger denotes significant differences between each condition at each time point (*P* < 0.05).

The mean baseline MEP amplitudes [standard deviation (SD)] were 0.89 (0.48) mV in iTBS, 0.99 (0.60) mV in cTBS, and 1.00 (0.65) mV in sham iTBS. The mean raw values did not differ significantly among the three protocols (mixed model one-way ANOVA, *F*_2, 141_ = 0.60, *P* = 0.551). A linear mixed model two-way repeated-measures ANOVA revealed a significant interaction between protocol and time (*F*_8, 658_ = 4.43, *P* < 0.001) and a main effect of protocol (*F*_2, 658_ = 40.66, *P* < 0.001) on the normalized MEP amplitudes while no main effect of time was found (*F*_4, 658_ = 0.57, *P* = 0.684) (Figure 2). *Post-hoc* tests revealed that the normalized MEP amplitudes of iTBS were higher at Post-0 (*P* = 0.002), Post-15 (*P* = 0.002) and Post-30 (*P* = 0.001) than that of iTBS at Pre. Moreover, the normalized MEP amplitudes of cTBS were significantly lower at Post-0 (*P* = 0.002) and Post-15 (*P* = 0.011) than that of cTBS at Pre. No significant differences were detected for sham iTBS. The comparison of the three protocols at Post-0 revealed that the normalized MEP amplitudes of iTBS were significantly higher than that of cTBS and sham iTBS (*P* < 0.001 and *P* = 0.001, respectively). At Post-15, the normalized MEP amplitudes of iTBS were significantly higher than that of cTBS and sham iTBS (*P* < 0.001 and *P* = 0.008, respectively). Moreover, the comparison of the three protocols at Post-30 showed that the normalized MEP amplitudes of iTBS were significantly higher than those of cTBS and sham iTBS (*P* < 0.001 and *P* = 0.016, respectively). No significant differences were recorded at Post-45. These results indicate that iTBS and cTBS have the potential to modulate the lower-limb corticospinal excitability.

### SICI and ICF

**Figure 3:**
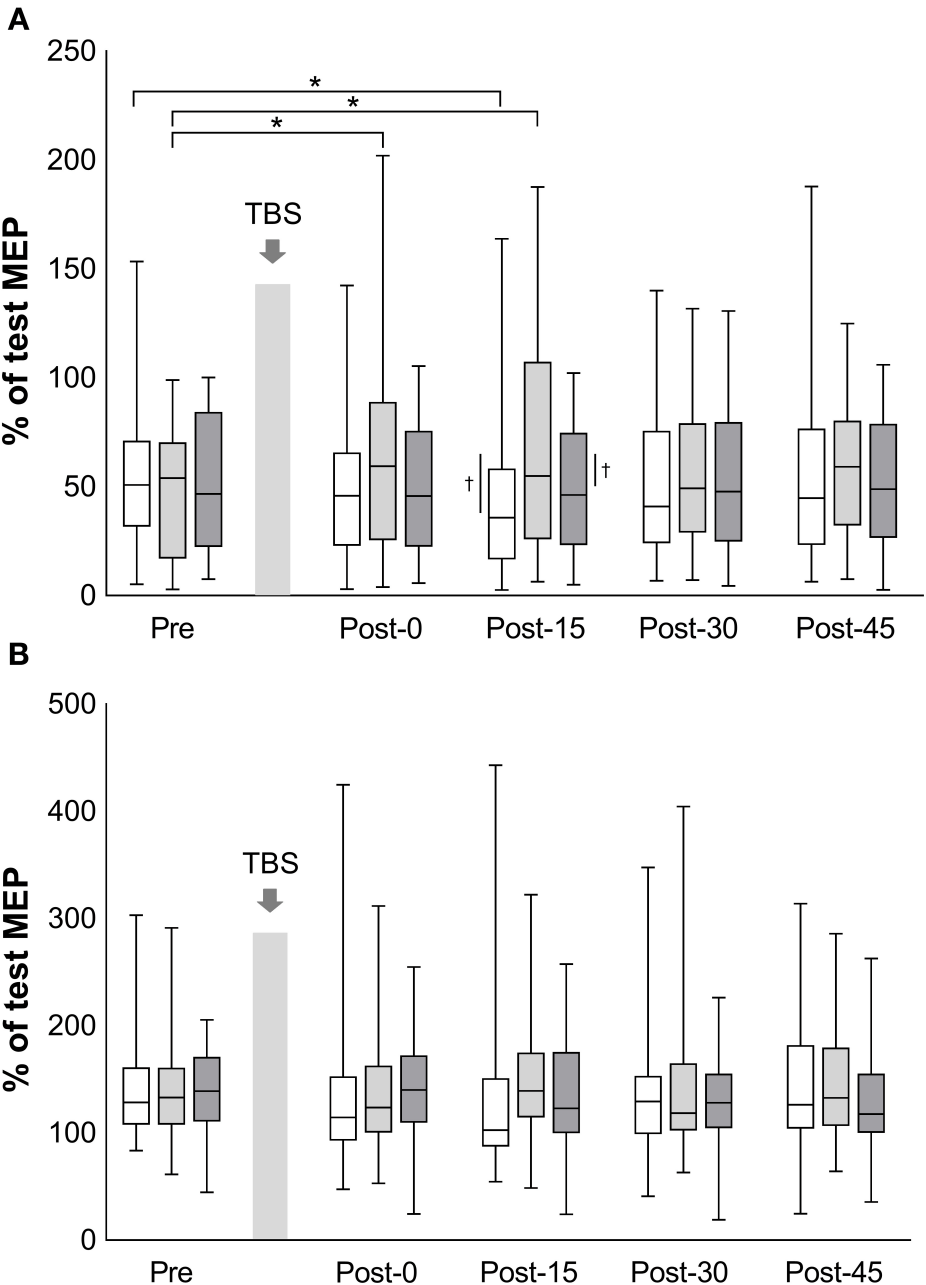
Effects of TBS on short-interval intracortical inhibition and intracortical facilitation. The values of short-interval intracortical inhibition (%) **(A)** and the values of intracortical facilitation (%) **(B)** are normalized to the amplitude of test MEP at each time point. White box plots, iTBS; light gray, cTBS; dark gray, sham iTBS. Median and interquartile ranges are represented by horizontal lines within boxes and whiskers (representing minimum and maximum values), respectively. The asterisk denotes significant differences between Pre and each time point within the conditions. The dagger denotes significant differences between each condition at each time point (*P* < 0.05).

The mean test stimulus intensity (range) of test stimulus was 62% (30–84) in iTBS, 63% (32–87) in cTBS, and 62% (32–88) in sham iTBS. The time courses of SICI and ICF are shown in Figure 3. The mean amount of SICI at the baseline (SD) was 52.9% (30.1%) in iTBS, 46.6% (28.5%) in cTBS, and 50.5% (28.9%) in sham iTBS. The baseline ICF (SD) was 145.3% (46.1%) in iTBS, 138.5% (44.0%), and 140.3% (38.3%) in sham iTBS. No significant differences were observed in the baseline condition among the three protocols (mixed model one-way ANOVA; SICI: *F*_2, 141_ = 0.58, *P* = 0.563; ICF: *F*_2, 141_ = 0.32, *P* = 0.727). For the results of all time points before and after TBS, a significant interaction was found for SICI (*F*_8, 658_ = 3.26, *P* = 0.001). There was also a significant main effect of protocol (*F*_2, 658_ = 10.08, *P* < 0.001), but no significant main effect of time (*F*_4, 658_ = 0.74, *P* = 0.568). No significant interactions (*F*_8, 658_ = 1.34, *P* = 0.22) or main effects (protocol: *F*_2, 658_ = 1.31, *P* = 0.27; time: *F*_4, 658_ = 0.27, *P* = 0.90) were observed for ICF. The SICI at Post-15 of iTBS was significantly stronger than that of iTBS at Pre (*P* = 0.045). In addition, the SICI of cTBS was significantly weaker at Post-0 (*P* = 0.002) and Post-15 (*P* < 0.001) than that of cTBS at Pre. No significant differences were recorded for the sham iTBS. We found that the iTBS was significantly stronger than cTBS at Post-15 (*P* = 0.001). Moreover, the cTBS was significantly weaker than the sham iTBS (*P* = 0.013) at Post-15. These results suggest that iTBS increases SICI in the lower-limb motor cortex, whereas cTBS has the opposite effect.

### Interindividual Variability in Response to TBS

A two-step cluster analysis showed two clusters for iTBS and cTBS in the present study. One cluster showed the results of a previous study that investigated the effects of each TBS on the upper-limb primary motor cortex (Huang et al., 2005), and the other showed no or opposite effects of each TBS. Based on a study (López-Alonso et al., 2014), we termed the cluster showing that the motor cortex excitability was modulated in the same direction as that reported in previous studies as “responders,” and the cluster showing that the excitability was not modulated in the same direction as “non-responders.”

Twenty-seven percent of the participants (13/48) exhibited a significant increase in MEP amplitude at Post-0 of the iTBS protocol, and 63% of the participants (30/48) showed a significant decrease in MEP amplitude after the cTBS protocol. Additionally, 73% of the participants (35/48) were classified as non-responders of the iTBS protocol, and 38% (18/48) were classified as non-responders of the cTBS protocol. Furthermore, 21% of the participants (10/48) were classified as responders of both TBS protocols. The mean % values of baseline MEP (SD) for each cluster at Post-0 were 125.5% (16.4%) for responders of the iTBS protocol, 104.1% (27.4%) for non-responders of iTBS protocol, 79.4% (13.5%) for responders of the cTBS protocol, and 106.4% (15.2%), for non-responders of the cTBS protocol. The normalized MEP amplitudes in the responders of iTBS protocol were higher than that in the non-responders of iTBS at Post-0 (*P* < 0.001). Moreover, the normalized MEP amplitudes in the responders of cTBS were significantly lower than that in the non-responders of cTBS at Post-0 (*P* < 0.001).

### Correlations Between TBS Response and Baseline Measurements

**Figure 4:**
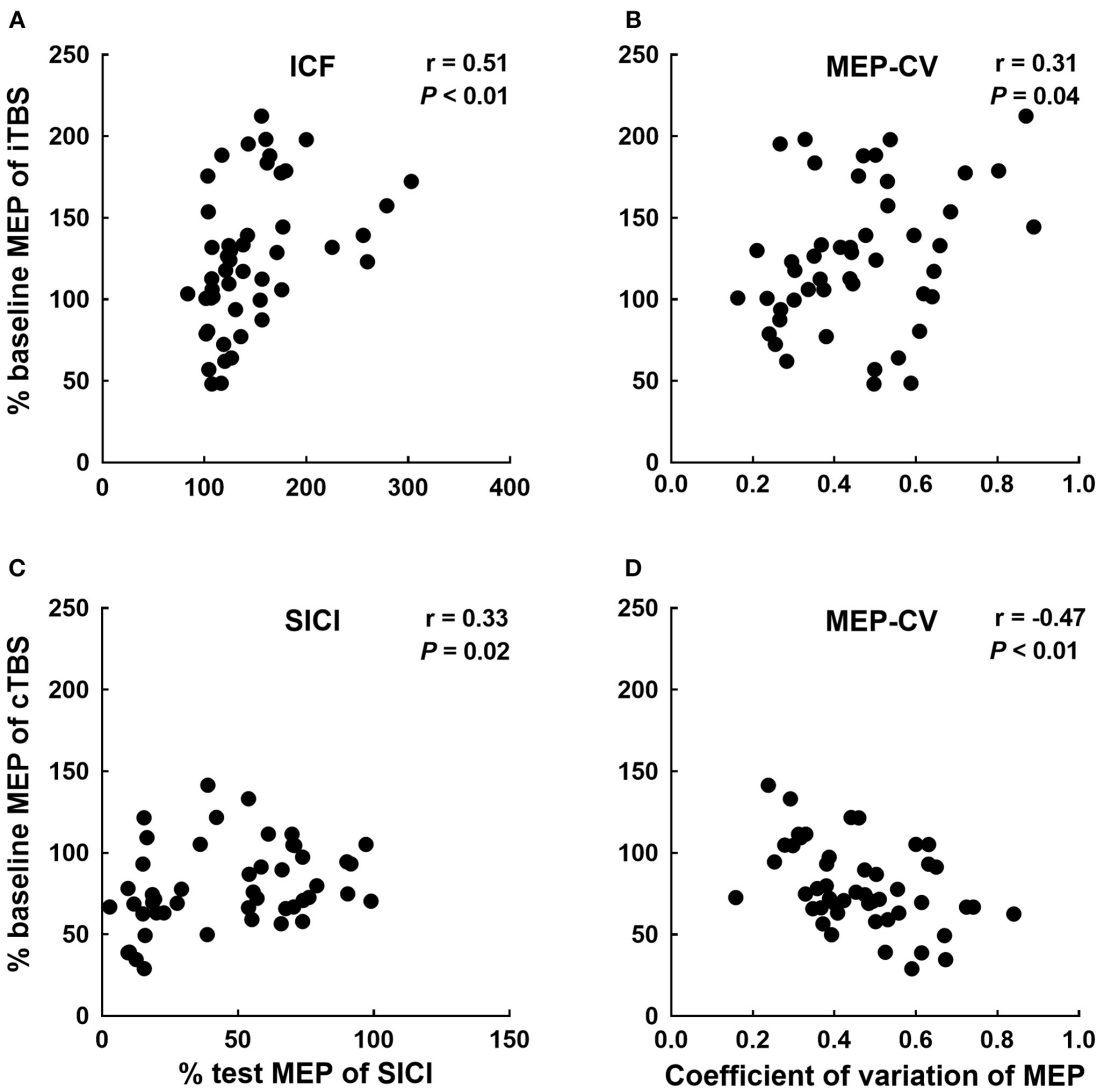
Significant correlations between each TBS response and neurophysiological factors. Correlations between the iTBS response (% of baseline MEP at Post-0) and ICF **(A)** and MEP-CV **(B)** at Pre. The correlations between the cTBS response (% of baseline MEP at Post-0) and SICI **(C)** and MEP-CV **(D)** at Pre.

A binary logistic regression analysis did not identify any factors affecting each TBS. Subsequently, we also calculated Spearman's rank correlation coefficients to evaluate the correlations between each TBS response for MEPs and baseline physiological measurements (Table 1). We detected significant correlations between the iTBS response and ICF and MEP-CV. Moreover, significant correlations were observed between the cTBS response and SICI and MEP-CV (Figure 4).

**Table 1:** Correlation between MEP response to each TBS and the physiological factors at the baseline and effects of each TBS.

	**iTBS**		**cTBS**	
**Physiological factors**	***r***	***P*-value**	***r***	***P*-value**
SICI	−0.234	0.109	0.330	0.022*
ICF	0.505	<0.001*	0.131	0.376
MEP-CV	0.305	0.035*	−0.470	0.001*
The slope of RC	0.087	0.555	−0.001	0.997
RMT	0.063	0.671	−0.233	0.110
AMT	0.264	0.070	−0.185	0.208

*The physiological factors are SICI, ICF, coefficient of variation of the MEP (MEP-CV), the slope of RC, RMT and AMT. The asterisks indicate a significant correlation between TBS response and the baseline physiological factors*.

## Discussion

This was the first study that applied TBS to the lower-limb motor cortex to examine its effects on intracortical excitability and the variability of effects on cortical excitability among individuals. This study showed that the application of iTBS to the lower-limb motor cortex increased corticospinal excitability up to 30 min, whereas the application of cTBS decreased corticospinal excitability up to 15 min. In addition, the individuals variability was large for iTBS and small for cTBS. We also showed that the TBS-induced corticospinal plasticity was related to cortical facilitation for iTBS, cortical inhibition for cTBS, and MEP variability for both before the intervention.

### Effects of TBS on Corticospinal Excitability

A meta-analysis on the effects of iTBS on the upper-limb motor cortex reported that corticospinal excitability increases significantly up to 30 min after the stimulation (Chung et al., 2016). Similar to that report, plastic changes in corticospinal excitability were observed here up to the 30 min time point after the application of iTBS to the lower-limb motor cortex. The suggested mechanism via which iTBS acts on the upper-limb motor cortex is that the iTBS selectively modulates the interneurons in layers 2 and 3 of the motor cortex, thereby inducing LTP-like synaptic plasticity and resulting in increased synaptic transmission (Hamada et al., 2013; Di Lazzaro and Rothwell, 2014; Huang et al., 2017). Our results showed that the increased corticospinal excitability lasted for the same time as did those reported previously for the upper-limb motor cortex. Therefore, a similar mechanism of action may be involved in the lower-limb motor cortex.

After cTBS, a decrease in corticospinal excitability was observed up to the 15 min time point after the stimulation. Conversely, a meta-analysis of the upper-limb motor cortex reported a decrease in the excitability up to 60 min after cTBS stimulation (Chung et al., 2016). The fact that the lower-limb motor cortex, which is the object of stimulation, is localized in a deeper layer compared with the upper-limb motor cortex (which likely affected the excitability) may explain the observation that the duration of the sustained effect was shorter. cTBS has been suggested to cause selective modification of the interneurons in layer 5 of the motor cortex, thus inducing LTD-like synaptic plasticity, which is involved in decreasing synaptic transmission efficacy (Hamada et al., 2013; Di Lazzaro and Rothwell, 2014; Huang et al., 2017). The stimulus intensity of cTBS in this study was ~61% (percentage for the maximum output of the TBS device), which was high compared with the ~39% used in a study that applied cTBS to the upper-limb motor cortex (Sasaki et al., 2018). This demonstrates that the upper-limb motor cortex is more easily stimulated compared with the lower-limb motor cortex. Additionally, cTBS selectively modulated the interneurons in layer 5, which was a relatively deep position within the cortex that consists of six layers. Furthermore, a previous study observed an intensity-dependent shift in the direction of the effects of cTBS applied over M1 (Doeltgen and Ridding, 2011). Thus, when applying cTBS to the lower-limb motor cortex, even if a higher stimulus intensity is used, an adequate stimulation input may not have been performed as the target stimulation site is located in an anatomically deep area of the lower-limb motor cortex. Furthermore, a previous study has reported that the stimulus intensity of cTBS affects the sustainability of the effect (Sasaki et al., 2018). Based on these reports and our results, differences in the anatomical sites of the lower-limb motor cortex and upper-limb motor cortex likely affected the duration of the effect.

In our study, the effects of iTBS on corticospinal excitability lasted longer than those of cTBS despite magnetic fields of the same strength. The simplest explanation for this result is that the stimulus pattern of TBS is an important determinant of the direction of the aftereffects following TBS (Suppa et al., 2016; Huang et al., 2017). It is assumed that the direction and the duration of TBS-induced plasticity on corticospinal excitability are determined by the amount and rate of Ca^2+^ influx generated by the TBS stimulus pattern (Huang et al., 2011, 2017; Suppa et al., 2016). In the iTBS paradigm, the rapid increases in the post-synaptic concentration of Ca^2+^ at the start of each 2-s train cause a greater production of facilitatory than inhibitory substances, e.g., levels of protein kinases (Huang et al., 2011). By contrast, in the cTBS paradigm, Ca^2+^ causes a slower-rising but larger increase in the amount of the inhibitory substance (Huang et al., 2011). Thus, the persistence of the aftereffects may depend on the changes in Ca^2+^ influx generated by the stimulus pattern of each TBS over the lower-limb and upper-limb motor cortices.

### Effects of TBS on SICI and ICF

In this study, a significant increase in SICI was observed only at 15 min after the iTBS stimulation. In the case of cTBS, the significant decrease in SICI lasted up to 15 min. Moreover, no significant changes were observed for ICF. The first report of TBS application to the upper-limb motor cortex revealed that TBS had long-lasting effects on SICI and ICF up to ~8–20 min (Huang et al., 2005); however, a meta-analysis reported no clear conclusions regarding the long-lasting effects of TBS on SICI or ICF (Chung et al., 2016).

It has been reported that the SICI circuits are differently activated because of anatomical differences between the upper- and lower-limb motor cortices (Di Lazzaro et al., 2001). The conditioning TMS pulse of the SICI in the upper-limb motor cortex selectively suppressed the I2 and later I-waves but not the I1-wave, while that in the lower-limb motor cortex resulted from clear suppression of the later waves (e.g., I3 and I4) (Di Lazzaro et al., 2001). Similar responses in SICI in the lower and upper limbs suggests that cTBS induces plasticity changes in SICI circuits parallel with that in the corticospinal excitability; however, the involved circuits may not be identical.

In contrast, no significant change in ICF was seen in our study. The original paper revealed that ICF did not change significantly after iTBS, while it was significantly reduced after cTBS (Huang et al., 2005). The explanation for these differences (i.e., the ICF changes were not clearly detected in this study) is unknown, but it may be because of the lower reliability of ICF measurements. ICF varies notably between tests; therefore, the re-test reliability of its measurement has been reported to be low compared with the measurements of MEP and SICI (Hermsen et al., 2016; Fried et al., 2017).

### Interindividual Variability in Corticospinal Excitability Changes Following TBS

The responsiveness to TBS over the upper-limb motor cortex was reported as being 52% for iTBS and 42% for cTBS (Hamada et al., 2013). However, in this study, the responsiveness to iTBS over the lower-limb motor cortex was 27%, which was low, while the responsiveness to cTBS was high, at 63%. By contrast, individual data show that 71% of the participants had a change of more than 100% of baseline MEP at Post-0 following iTBS (please see the Supplementary Material showing individual MEP changes). These results indicate that the significant group-averaged changes in normalized MEP revealed by ANOVA may not have the same meaning regarding the facilitatory effects of iTBS on corticospinal excitability as that of the cluster analysis. Previous studies used the cluster analysis or subgrouping techniques (e.g., classification based on <1/>1 criteria, classification based on arbitrary percentage change from baseline criteria, etc.) to identify responders and non-responders in NIBS research (Pellegrini et al., 2018). This systematic review suggests that cluster analysis is an effective approach for classifying responders and non-responders within the analysis (Pellegrini et al., 2018). In addition, cluster analysis has been reported to produce the fewest number of false positive classifications for several different classification methods (van de Ruit and Grey, 2019). Therefore, our results will likely be useful for defining responders in future works. One caveat is that the sample size of the present study is too small to determine the criteria for responders after TBS, and thus additional work must be conducted with larger sample sizes.

It remains unclear why our results were different from those of a previous study. It is known that the sensitivity of the respective interneurons that were the stimulation targets of iTBS and cTBS was different between the studies. Late I-waves that are generally believed to be generated by multiple synaptic neural circuits and have a high evoked threshold are modulated by iTBS, whereas I1-waves generated by single synaptic neural circuits with a low evoked threshold are suppressed by cTBS (Di Lazzaro et al., 2001). Several lines of evidence suggest that neural elements activated by TMS differ between lower-limb and upper-limb motor cortices (Priori et al., 1993; Nielsen et al., 1995; Houlden et al., 1999; Terao et al., 2000). Predominant descending volleys elicited by TMS over the lower-limb motor cortex have always been the I1-wave volleys, regardless of the current direction in the brain (Terao et al., 2000). The difference in these neural elements activated by TMS is likely reflected in differences in responsiveness to iTBS and cTBS between lower and upper limbs.

Another possibility is the involvement of interhemispheric cortical interactions. The motor cortices of two hemispheres are thought to interact primarily through the corpus callosum (Wahl et al., 2007). Considering both lower-limb motor cortices are proximally located, it is difficult to apply TBS to the motor cortex of one hemisphere without stimulating the other, especially at higher stimulus intensity (Huang et al., 2018; Lin et al., 2019). Additionally, the interaction between synergistic and antagonist cortical representations of arm muscles affects the plasticity induced by TBS (Mirdamadi et al., 2015, 2016). Therefore, the inducibility of LTP-like or LTD-like synaptic plasticity is different in different motor cortical representations. Thus, the propagation of TBS to the non-targeted hemisphere may result in interhemispheric interactions and affect responsiveness to TBS, even though the interhemispheric interactions in the motor cortices of lower-limbs are less understood (Charalambous et al., 2016).

However, the varying responses to TBS could be related to the difference in the motor function role between hands (mainly fine motor control) and legs (mainly cyclic movement, such as a gait). The different responsibilities in the brain may affect the responsiveness of synaptic plasticity induced by TBS (Li et al., 2019). A study reported that TBS induces significant cortical plasticity in hand muscles, whereas no significant response was observed in arm muscles (Martin et al., 2006). This further supports the fact that the inducibility of LTP-like or LTD-like synaptic plasticity is different in different motor cortical representations.

### Relationship Between the Effects of TBS and Neurophysiological Parameters Recorded Before the Intervention

Similar to a report of the application of cTBS to the upper-limb motor cortex (Hordacre et al., 2017), the changes in MEP observed after iTBS and the MEP-CV recorded before the intervention were significantly correlated in the present study. In addition, the changes in MEP recorded after cTBS and the MEP-CV recorded before the intervention were significantly inversely correlated. The intrinsic oscillatory properties of the cortex have been reported to be related to the variability in MEP amplitude (Sauseng et al., 2009; Schulz et al., 2014). Moreover, the direction of the TBS effect was reported to depend on a change in Ca^2+^ influx in the post-synaptic membrane, which is governed by the pulse frequency (Huang et al., 2017). Thus, the individual characteristics of the intrinsic oscillatory properties that were evaluated via MEP variability likely affected the effects of TBS on corticospinal excitability.

The correlation between the changes in MEPs observed after iTBS and ICF recorded before the intervention, and the changes in MEPs observed after cTBS and SICI recorded before the intervention are novel findings that have not been reported to date. The original TBS article suggests that TBS modulates synaptic plasticity in the circuits under the TMS coil, involving corticospinal excitability for MEPs, inhibitory circuits for SICI, and facilitatory circuits for ICF simultaneously (Huang et al., 2005). Review articles further suggest that both GABAergic and glutamatergic neurotransmission play a crucial in the induction of plasticity by TBS (Suppa et al., 2016; Huang et al., 2017; Li et al., 2019). It is also well-known that SICI is mediated by GABAergic interneurons and ICF could be mediated by glutamatergic interneurons (Ziemann et al., 2015). Therefore, the responsiveness of SICI and ICF by TMS assessment at baseline may be related to the changes in MEPs following TBS and be responsible for the interindividual variability of lower-limb motor cortical plasticity.

### Limitations

The current study recruited healthy young humans. Because age affects TBS and other NIBS-induced synaptic plasticity (Suppa et al., 2016; Huang et al., 2017), caution must be exercised when making inferences using the present results from healthy young subjects to other populations with different biological characteristics. Additionally, other factors, such as genetics, sex, and anatomy of neural circuits, play crucial roles in the induction of plasticity following NIBS (Suppa et al., 2016; Huang et al., 2017). Future research is warranted to investigate the effects of TBS on the lower-limb motor cortex in other healthy populations or patients with neurological diseases such as lower-limb paralysis.

Furthermore, we did not assess the cortical excitability of the soleus muscle in the current work. Previous studies reported that the overlapping cortical representations with proximal antagonist muscle affect each TBS (Mirdamadi et al., 2015, 2016). Similarly, TBS to the motor cortex area of soleus muscleareas that overlaps with TA muscle may affect our results. Therefore, in future studies, we would like to assess the effects of TBS on soleus MEPs.

## Conclusion

The current study provides evidence of the induction of an increase in corticospinal excitability after the application of iTBS to the lower-limb motor cortex. However, the changes observed were the low responsivity compared with the application of iTBS to the upper-limb motor cortex (Hamada et al., 2013). Conversely, cTBS decreased corticospinal excitability and had a high responsivity. Moreover, the corticospinal excitability changes that were induced by iTBS were correlated with ICF before the intervention, and the changes induced by cTBS were correlated with SICI, suggesting that TBS modulates different circuits under the TMS coil simultaneously and the change in ICF might contribute to the effect of iTBS and that in SICI might contribute to the effect of cTBS on MEPs. These findings contribute to the understanding of interindividual variability in response to TBS in the motor cortex of the lower limb, and helpful for further applications of TBS for the lower-limb motor cortex in patients with neurological disorders.

## Data Availability Statement

The raw data supporting the conclusions of this article will be made available by the authors, without undue reservation.

## Ethics Statement

The studies involving human participants were reviewed and approved by Ethics Review Board of the Yamagata Prefectural University of Health Sciences. The patients/participants provided their written informed consent to participate in this study.

## Author Contributions

NK and TY conceived the study, designed the research, managed the project, analyzed, interpreted the data, drafted, and wrote the manuscript. NK, SY, and TY recruited the participants and collected data. ST made the program for data collection and analysis. TK, DK, SN, and Y-ZH revised the manuscript. TY procured funding. All authors approved the final version of the submitted manuscript.

## Conflict of Interest

The authors declare that the research was conducted in the absence of any commercial or financial relationships that could be construed as a potential conflict of interest.

## Abbreviations

AMT: active motor threshold
ANOVA: analysis of variance
cTBS: continuous theta burst stimulation
EMG: electromyography
GABA: g-amino butyric acid
ICF: intracortical facilitation
I-wave: indirection wave
ISI: interstimulus interval
iTBS: intermittent theta burst stimulation
LTP: long-term potentiation
LTD: long-term depression
MEP: motor evoked potential
MEP-CV: coefficient of variation of motor evoked potential
NIBS: non-invasive brain stimulation
NMDA: *N*-methyl-D-aspartate
RC: recruitment curve
RMT: resting motor threshold
SICI: short-interval intracortical inhibition
TA: tibialis anterior muscle
TMS: transcranial magnetic stimulation.
