# Supplementary material for: Interindividual Variability of Lower-Limb Motor Cortical Plasticity Induced by Theta Burst Stimulation
Source: Front Neurosci. 2020 Nov 13;14:563293. doi: 10.3389/fnins.2020.563293 (PMC7691321; doi:10.3389/fnins.2020.563293)
Supplement: Supplementary file 1 [file Data_Sheet_1.pdf]

Additional information

Supplemental table

	Stimulus intensities of TBS (%)	MEP amplitudes of each TMS intensity (mV)							Coefficient of variation of the MEP (arb. u.)
		80%	100%	120%	140%	160%	180%	200%	
iTBS	61.1 (8.6)	0.19 (0.09)	0.32 (0.14)	0.71 (0.39)	1.20 (0.59)	1.62 (0.77)	1.92 (0.77)	2.03 (0.75)	0.46 (0.17)
cTBS	61.4 (9.6)	0.21 (0.11)	0.31 (0.16)	0.73 (0.44)	1.16 (0.63)	1.58 (0.67)	1.84 (0.65)	1.94 (0.63)	0.47 (0.15)
sham iTBS	61.4 (8.0)	0.20 (0.10)	0.33 (0.16)	0.69 (0.36)	1.18 (0.63)	1.57 (0.71)	1.82 (0.72)	1.99 (0.68)	0.44 (0.17)

Values represent mean (standard deviation).

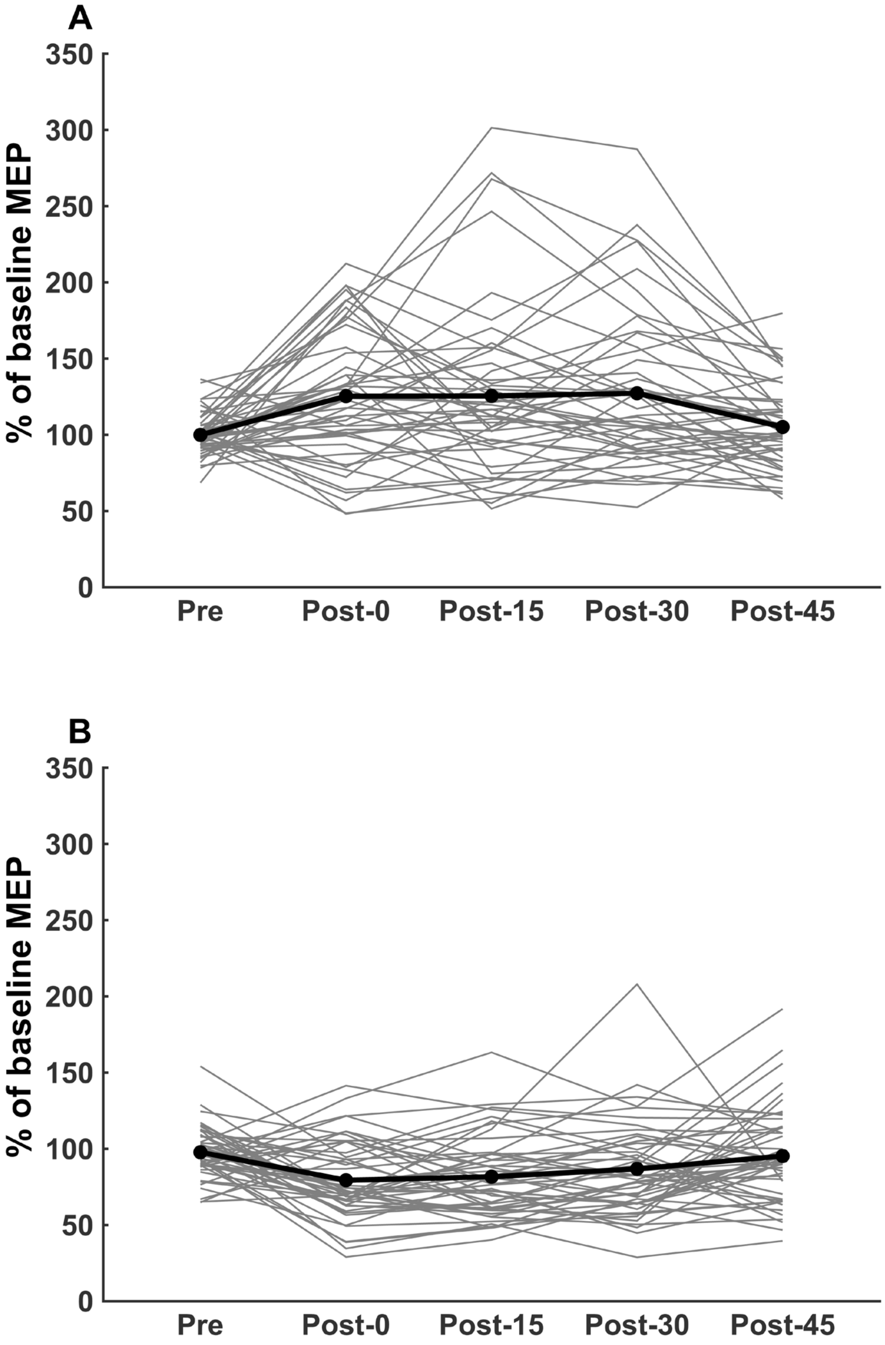

Supplemental Figure. The effects of TBS on corticospinal excitability in individual participants.

The changes of corticospinal excitability are normalized with respect to the amplitude of MEP at baseline following iTBS (A) and cTBS (B). The thin grey line shows the individual time courses of excitability changes, and the thick black line shows the average values at each point.
